# Antibacterial activity of silver-killed bacteria: the "zombies" effect

**DOI:** 10.1038/srep09555

**Published:** 2015-04-23

**Authors:** Racheli Ben-Knaz Wakshlak, Rami Pedahzur, David Avnir

**Affiliations:** 1 Institute of Chemistry and the Center for Nanoscience and Nanotechnology, the Hebrew University of Jerusalem, Jerusalem 91904, Israel; 2 Department of Environmental Health, Hadassah Academic College, Jerusalem 91010, Israel

## Abstract

We report a previously unrecognized mechanism for the prolonged action of biocidal agents, which we denote as the zombies effect: biocidally-killed bacteria are capable of killing *living* bacteria. The concept is demonstrated by first killing *Pseudomonas aeruginosa* PAO1 with silver nitrate and then challenging, with the dead bacteria, a viable culture of the same bacterium: Efficient antibacterial activity of the killed bacteria is observed. A mechanism is suggested in terms of the action of the dead bacteria as a reservoir of silver, which, due to Le-Chatelier's principle, is re-targeted to the living bacteria. Langmuirian behavior, as well as deviations from it, support the proposed mechanism.

Antimicrobial agents play an essential role in the control of infectious diseases and of the spread of pathogens. In addition to the immediate action of reducing bacterial loads, it is highly desirable for an antimicrobial agent to have long-term effectiveness^1^,^2^, thereby preventing bacterial re-colonization and proliferation. Incorporation of antimicrobial agents within sustained-release delivery systems that enable their continuous use is one of the most investigated methods to prolong antimicrobial activity^3^,^4^. Prolonged activity is also characteristic of biocidal metals such as silver and copper that slowly release their cations, trace amounts of which are toxic to bacteria^5^,^6^,^7^. Considerable experimental evidence has been accumulated on the prolonged effectiveness of antimicrobial metals through the slow-release of their cations, preventing contamination of wounds^1^,^8^,^9^, biomedical devices^5^,^10^, and textiles^11^.

Here we report a route for the prolonged action of biocidal agents, silver in our case, which, to the best of our knowledge has not been previously recognized, and which for reasons that will shortly be detailed, we denote as the zombies effect: Bacteria which were killed by silver show significant biocidal activity towards *viable* population of the same bacterium. The origin of this phenomenon, as we show here, is in two characteristics of the metal-induced biocidal action: First, the metallic species are not deactivated by the killing mechanism, and therefore can carry on their biocidal effect repeatedly; and second, the dead bacteria serve as an efficient sustained release reservoir for releasing the lethal metallic cations for further action against other live bacteria; this is a manifestation of Le-Chatelier's principle that operates here, as is explained below.

## Methods

### Chemicals

AgNO_3_, chlorhexidine digluconate, 20% (w/v), HNO_3_, ~70% (w/w) were purchased from Sigma-Aldrich. Chemicals for the preparation of the neutralization solution: sodium thioglycolate, sodium thiosulfate, lecithin, were purchased from Acros Organics; Tween 80 (polyethylene glycol sorbitan monooleate) was purchased from Fluka.

### Bacteria

Wild-type *Pseudomonas aeruginosa* PAO1 strain was kindly provided by Prof. E. Banin, Bar-Ilan University, Israel.

### Evaluating the antibacterial effect of killed bacteria

Three overnight cultures were prepared by seeding single colonies into Luria Bertani (LB, 25 mL) broth at 37°C with shaking. These overnight cultures were then washed three times by centrifugation (10 min, 4800 rpm, at RT) and re-suspended in HEPES buffer (0.04 M, pH 7.4). Each washed pellet was re-suspended in HEPES (5.0 ml) and the three suspensions were combined. The combined suspension was brought to an optical density (OD_590 nm_) of 0.6 which corresponds to *ca.* 2·10^8^ colony forming units (CFU)/ml. The bacterial suspensions (5.0 ml) were added to sterile centrifuge tubes containing solutions of AgNO_3_ (5.0 ml) at increasing concentrations (1, 2, 5, 15 and 20 ppm). The tubes were stirred in an incubated shaker at 30°C under dark conditions for 6 h. Then, aliquots (0.8 ml) from the tubes were neutralized by dilution (1:1) with a neutralizing solution (0.2%w/w sodium thioglycolate, 1.9% w/w sodium thiosulfate, 1% w/w Tween 80, 1.4% w/w lecithin) for 5 min. After neutralization, the samples were serially diluted (10 fold) in phosphate buffer saline (PBS) and pour–plated with nutrient agar into Petri plates. The plates were incubated at 37°C for 48 h and the bacterial colonies were counted; the killing of 99.9% of the initial bacterial population in all treated samples was ensured. The silver treated bacterial suspensions were then centrifuged (10 min, 4800 rpm, RT) and the pellets were re-suspended (OD_590 nm_ of 0.3, 10 ml). The supernatants of the pellets were filtered using 0.2 μm syringe filters and a fresh concentrated bacterial suspension was added, which brought the optical density of the combined suspension to (OD_590 nm_) to 0.3. The tubes were incubated in an incubated shaker at 30°C under dark conditions for 6 h. Then, aliquots (1.0 mL) from all suspensions were neutralized, serially diluted, pour-plated, incubated and enumerated as described above. The bactericidal experiments were repeated two to three times and the mean log reduction values (log(N_t_/N_0_) where N_t_ = bacterial concentration at time t and N_0_ = bacterial concentration at time 0) for the various treatments were calculated. Evaluation of the effect of the chlorhexidine (CH)-killed bacteria towards viable bacteria was carried out by exposing the viable bacteria to the CH-killed bacteria (initially killed with a 20 ppm CH solution) and their supernatant solutions for 24 h.

#### Heat-treated bacteria control test

Examining the effect of heat-treated bacteria towards viable bacteria was carried out similarly as described above, but instead of the treatment with silver nitrate solutions, the initial bacterial suspension was autoclaved in 121°C for 10 minutes and then viable bacteria were exposed to the heat-killed bacteria for 24 h.

#### Silver adsorption in the bacterial cells

ICP-MS elemental analysis was used to measure the Ag^+^ concentrations present in silver-treated bacteria. The bacterial suspension was prepared and treated with silver nitrate solutions as described in the antibacterial efficacy experiment of killed bacteria. After 6 h of exposure to silver nitrate solutions, the bacterial suspensions were filtered with 0.2 μm syringe. Aliquots (0.5 ml) from the filtrated solutions were diluted with 1 M of distilled HNO_3_ solution (4.5 ml) and measured. Control silver nitrate solutions were prepared at the same concentrations with no bacteria were diluted in 1 M distilled HNO_3_ and measured as well. The Ag^+^ concentrations were evaluated using a calibration curve made by measuring standard solutions of AgNO_3_ prepared in 4.0 mM HEPES, 1 M distilled HNO_3_ solution at concentrations ranging from 0.020 to 2 ppm. The concentrations of the adsorbed Ag^+^ were calculated by subtracting the measured silver concentrations in bacterial supernatants from these in the control silver solutions.

#### TEM sample preparation and instrumentation

Control and silver-treated cells of *P. aeruginosa* were separated from their solution by centrifugation and re-suspended in 1.0 mL PBS. Then, the cells were fixed, post-fixed, dehydrated, embedded in epoxy resin, and thin sectioned as described elsewhere^31^. Optical density measurements were carried out with a Hewlett-Packard 8452 A diode-array UV-vis spectrophotometer. Ag^+^ concentrations and were measured with Agilent 7500 cx inductively coupled plasma mass spectrometer ICP-MS for trace metal analysis. TEM and STEM imaging were carried on a Tecnai 12 electron microscope (FEI) and Tecnai F20 G^2^ (FEI) high resolution transmission scanning electron microscope.

## Results and Discussion

**Figure 1:**
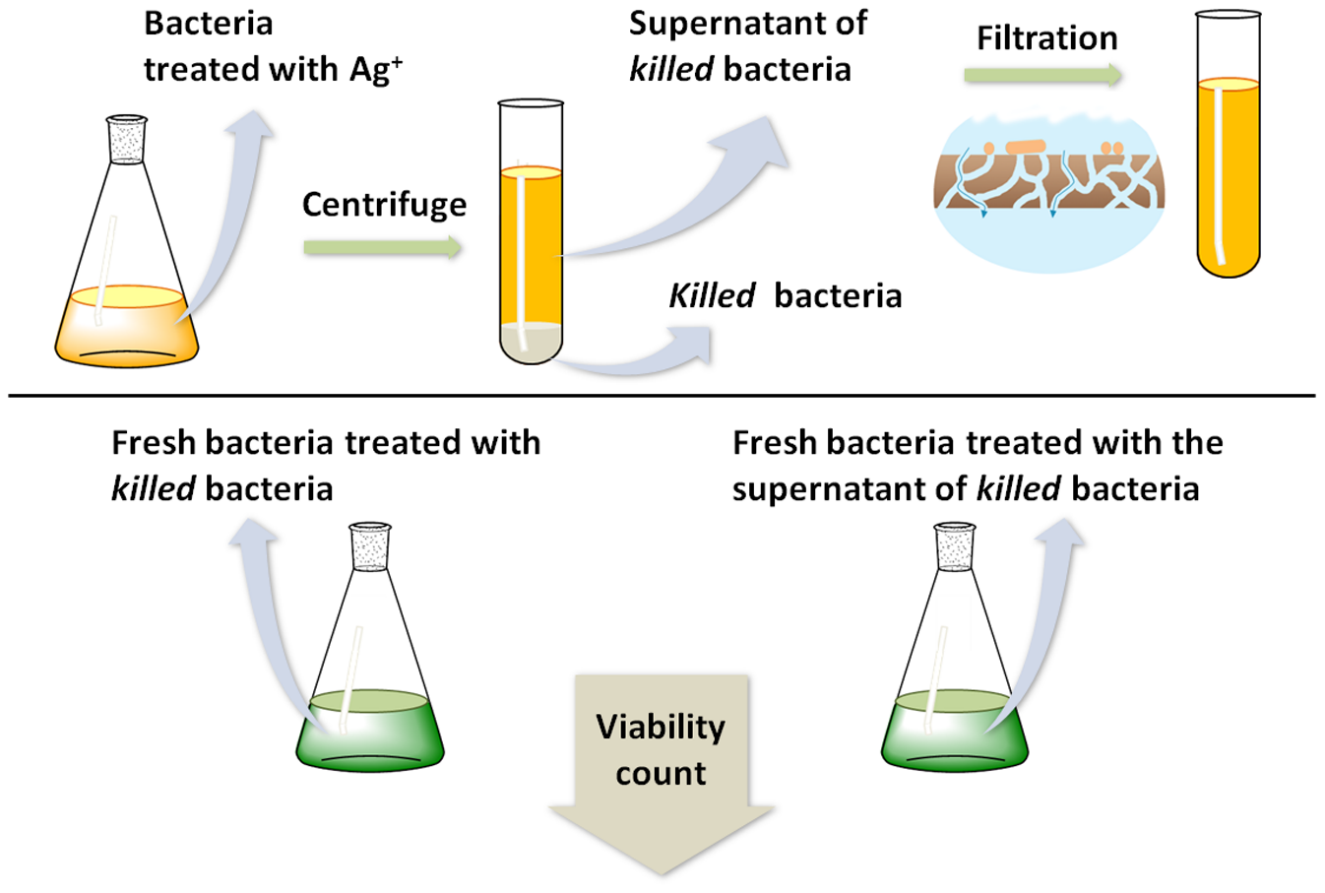
Overview of the zombies effect experiment. Figure created by R.B.-K.W.

The outline of the experiment is the following: Bacterial cells of the opportunistic pathogenic bacteria *Pseudomonas aeruginosa* PAO1 (viable bacterial population of *ca.* 10^8^ CFU/ml) were exposed to silver nitrate solution, a well-known primary antibacterial agent, at a concentration sufficient for killing the bacteria. The dead bacteria were separated and thoroughly cleansed from their supernatant medium by centrifugation and filtration (Figure 1); then, a fresh viable bacterial culture of *P. aeruginosa* of *ca.* 10^8^ CFU/ml was exposed to the dead bacteria and after 6 h of exposure, bacterial viability was enumerated.

**Figure 2:**
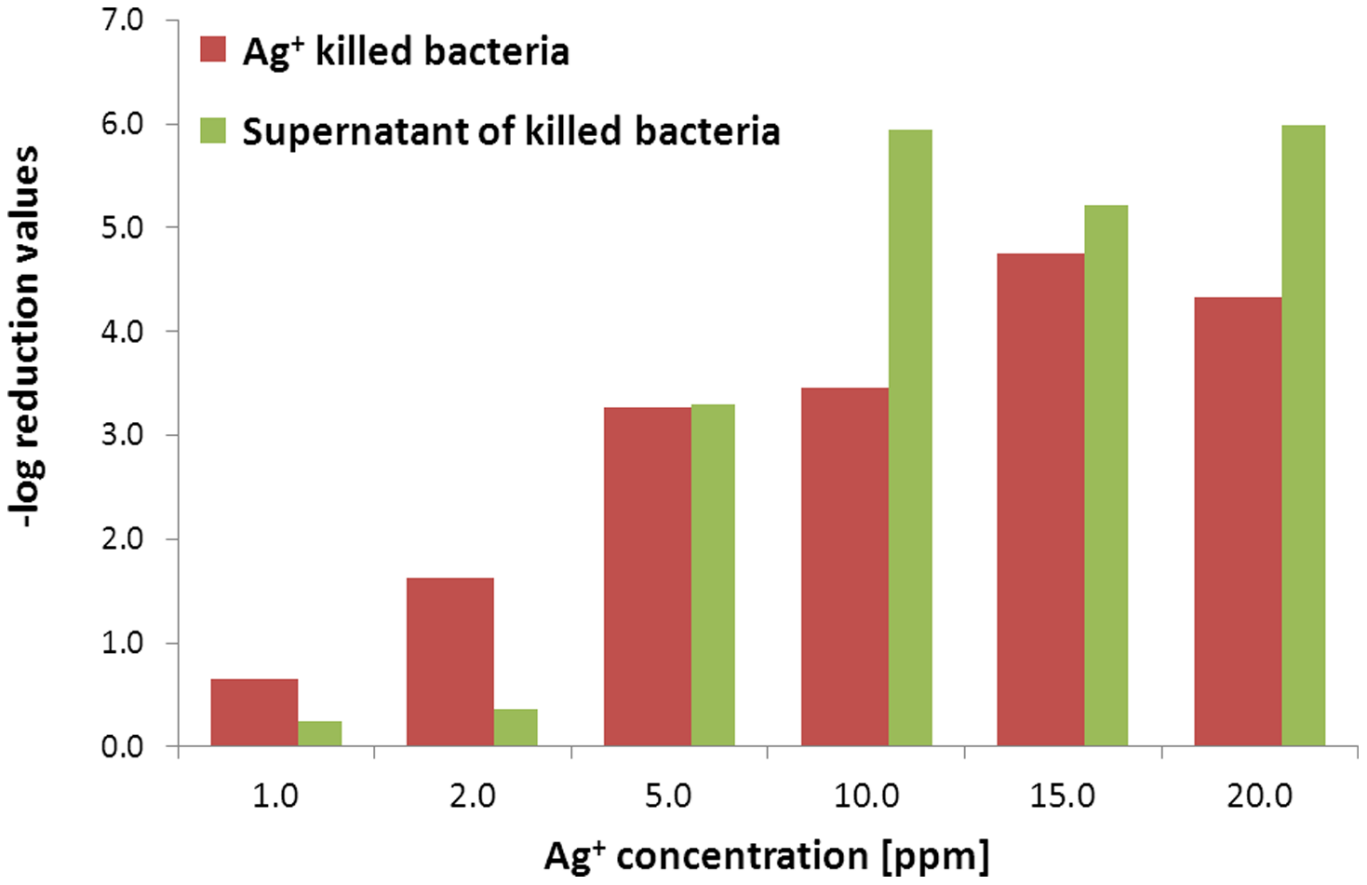
Shown is the bactericidal effect of silver-treated *P. aeruginosa* cells towards fresh viable cells at increasing silver nitrate concentrations, after 6 h of exposure. Green: The antibacterial activities of the supernatant solutions of the initial kill. (Error range: ~10% of the log reduction values). Red: The antibacterial activity of killed bacteria.

Figure 2 summarizes the results of the antibacterial activity of the silver-killed *P. aeruginosa* cells towards the suspension of the living cells: Two types of biocidal activity measurements are described there, as a function of the amount of the Ag^+^ initially used to form the zombies: In red are the biocidal activities of the dead bacteria which were killed by the amount of Ag^+^ indicated on the x-axis; in green are the biocidal activities of the Ag^+^-containing supernatant solutions, separated from the killed bacteria (Figure 1). It is seen that at all Ag^+^ concentrations the dead bacteria act as biocidal agents, reaching a maximum killing efficiency of 5 orders of magnitude (that is, 99.999% of the of *P. aeruginosa* are killed by the zombies). It is also seen that the killing efficiency increases sharply with concentration at the lower concentrations of the initial Ag^+^, and then more smoothly; this behavior is a telltale clue for the underlying mechanism of this phenomenon, and we return to it later on. Figure 2 strongly indicates that not only does silver persist within the dead cells, but that it is an available source for further biocidal activity on viable cells. The activities of the supernatant solutions which had been separated from the zombies were measured as well, and provide an additional indicator to the proposed mechanism. For this purpose note that with the increase in Ag^+^ concentrations, these solutions (green blocks) first show a weaker biocidal activity compared to the killing power of the zombies (red), then become equal to it (at 5.0 ppm), and then tend to be somewhat higher. The interpretation of this behavior is that at low Ag^+^ concentrations, most of the metal is chelated within the bacteria, that the capacity of that chelation has a limit, and as that limit is approached, the activity of the supernatant solution must increase, as it does indeed.

To rule out the possibility that the biocidal action is due to bacterial residues, we examined the effect of heat-killed bacteria on a viable population. It was found that the bactericidal effect is negligible, below the limit of detection. Thus, the dead bacteria do not account for the large biocidal effect of the zombies: their antibacterial activity is therefore related to their being a reservoir of silver.

**Figure 3:**
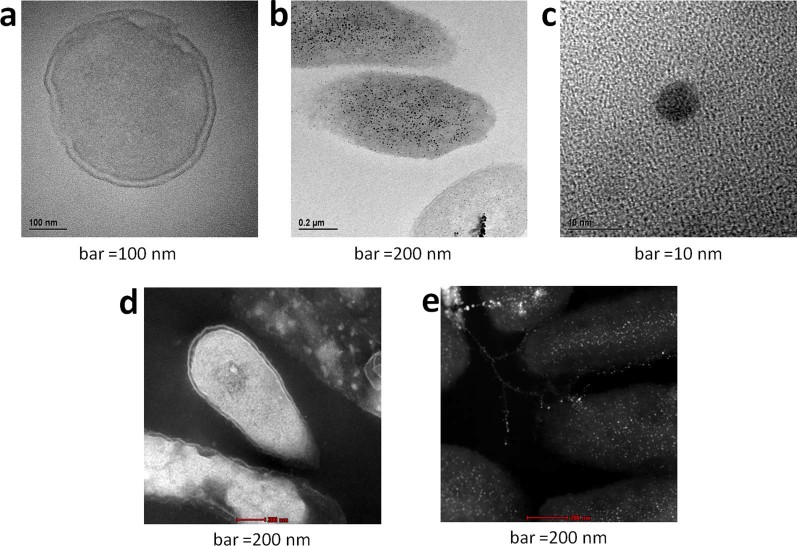
TEM (bright background) and STEM (dark background) of *P. aeruginosa* before (a and d) and after (b, c and e) treatment with silver; the black (b) and white (e) granules represent silver deposition which account for the zombies biocidal action.

Indeed, TEM and STEM images of cross sections of bacteria before (Figure 3(a) & (d)) and after (Figure 3(b), (c) & (e)) treatment with silver nitrate clearly show the accumulated silver as small nanoparticles, 5–10 nm in size, evenly distributed throughout the bacterium's cross section. A closer look at a single nanoparticle (Figure 3(c)) reveals lattice fringes with spacing characteristic of silver metal (2.36 Å, atomic planes {1,1,1}^12^), which is formed through the reduction of silver cations by the reducing environment of the living cell (it was suggested that reductive enzymes and reducing sugar molecules serve as reducing agents for such incorporated metal cations^13^).

**Figure 4:**
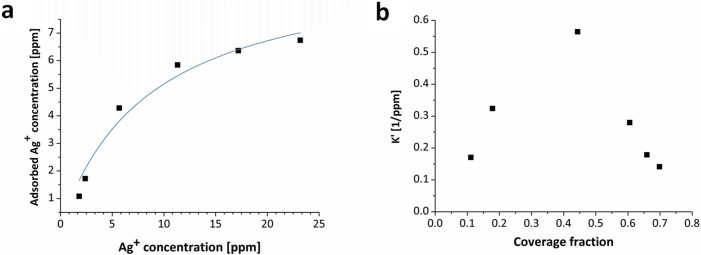
(a) Ag^+^ adsorption isotherm onto *P. aeruginosa* cells and its fit to Langmuir's equation. (b) The results of the more accurate Fireman-Shoresh *et al*^18^ analysis of the dependence of the adsorption equilibrium constant, *K*′, on coverage.

Using ICP-MS, the adsorption isotherm of Ag^+^ was determined, and is shown in Figure 4(a). Since the interaction of Ag^+^ with the cell components is strong and reversible, Langmuir-type analysis of the isotherm (Figure 4(a)) is relevant; here, the bacterium molecular components - sulfhydryl groups in various proteins, DNA and RNA^14^,^15^,^16^ - represent the adsorbent and Ag^+^ the adsorbate (we note that it is a common practice to evaluate biosorption of metal ions on microorganisms with Langmuir's model^17^). Indeed, as seen in Figure 4(a), despite the complexity of the system there is a good fit to Langmuir's isotherm equation (eq. 1),
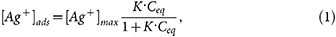
where [*Ag^+^*]*_ads_* is the Ag^+^ adsorbed equilibrium concentration, [*Ag^+^*]*_max_* is the maximum capacity of adsorption at equilibrium concentration, *K* is Langmuir's constant, and *C_eq_* is the Ag^+^ equilibrium concentration in the solution. Remarkably, the maximum coverage value calculated from this equation is in good agreement with the 5–10 ppm concentrations range of Figure 2: 9.6 ppm of Ag^+^, which is 96·10^14^ grams of silver per Colony Forming Unit (CFU) or, using Avogadro's number, 5·10^8^ silver atoms per CFU. Figures 2 & 4, corroborate each other.

Note again that at low Ag^+^ concentrations (below 5 ppm) the equilibrium favors attachment to the cell over the solubilization within the bacterial test medium, and therefore the dead cells are those capable of further bacterial inactivation. When the available cellular binding sites gradually become saturated with Ag^+^ (at concentration ~ 10 ppm), most of the excess Ag^+^ remains in the liquid medium, which leads to bactericidal action of both the dead-bacteria and the test medium (the supernatant). As indicated above (Figure 3) the adsorbed silver cations undergo - at least partially - reduction to metallic silver, and thus the reservoir is composed of both complexed and metallic silver. The presence of new, viable bacteria then acts as new, unoccupied adsorption sites for silver, and the equilibrium of silver between the reservoir and the liquid, is shifted, according to Le-Chattelier principle from the dead bacteria to the new viable ones:

- Ag^+^_(aq)_ + bacterium → Ag-killed bacterium
- Ag-killed bacterium ⇆ Ag^+^_(aq)_ + killed bacterium
- Ag^+^_(aq, from _eq. 2_)_ + viable bacterium → Ag-killed bacterium

In this context we recall that the migration of ions from one solid to another affected by equilibrium shifts is a known phenomenon, demonstrated, for instance, with *cis*-platin and thiol-modified sol-gel particles^18^, and with acid-base interactions between solids at distance^19^. It is also in order to comment here on the heterogeneity of the adsorbent (the various complexing sites) and on the application of Langmuir's model for such cases: heterogeneity is not an underlying feature of Langmuir's analysis, which assumes homogeneous adsorption sites. Therefore, simple Langmuir analysis provides an average picture. A more accurate analysis has to take into account the actual coverage-dependent behavior, which we have carried out by applying the analysis developed by Fireman-Shoresh *et al* for such situations^18^. In this analysis the coverage dependent adsorption equilibrium (*K*′, eq. 2) is represented by:
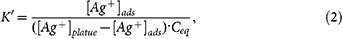
where [*Ag^+^*]*_ads_* refers to the coverage of Ag^+^, ([*Ag^+^*]*_plateau_* [*Ag^+^*]*_ads_*) represents the unoccupied sites, and *C_eq_* is the concentration of Ag^+^ in the solution at equilibrium. The dependency of the equilibrium constant on the coverage is shown in Figure 4(b). It is seen that *K*′ increases with coverage until a coverage fraction of 0.4 is reached. Following other studies of biosorption of metal cations^17^, we believe that this region of the graph represents a feed-back loop where the partial reduction of the adsorbed silver cation takes place on the growing metallic nanoparticles within the microbial cell. It is also seen in Figure 4(b) that beyond coverage fraction of 0.4 there is a decrease in the equilibrium constant representing the decrease in the amount of high-energy adsorption sites, and suggesting that the nanoparticles growth reached its final size due to the stabilization of capping biomolecules such as proteins^20^,^21^,^22^.

In conclusion, a new mechanism of significant residual effectiveness of antimicrobial agents was presented, which takes into account the availability and persistence of antimicrobials within inactivated bacterial cells, and the reversibility of their attachment to cellular components. We are not ready yet to answer the intriguing question of the relevance of this observation to wound healing applications. However, we allow ourselves a certain degree of optimism from that point of view, because like any other antibiotic compound, silver can be both bacteriostatic and bactericidal, depending on concentration and on the local environment^23^. This is true for wounds as well, that is, given the adequate dose of silver, bacterial infections of wounds can be treated to the level of almost total eradication of the pathogenic bacteria^24^. Very recently, Said et al showed^25^ that Ag^+^ is both bacteriostatic and bactericidal in growth medium at comparable silver concentrations, and that in simulated wound fluid, bactericidal activity is still possible but at higher concentrations. It follows therefore that prolonged action of silver ion releasing formulations^26^,^27^ benefit, at least in part, from the phenomenon we describe here. We also believe that this phenomenon is potentially general, and may exist in other antibacterial agents, particularly in those that are stable and undergo minimal degenerative transformations (quaternary ammonium compounds, copper, and metal oxides). Indeed, preliminary observations with wild-type *Pseudomonas aeruginosa PAO1* killed with chlorhexidine (CH), an antiseptic agent well known for its excellent persistence effect^27^,^28^, support this hypothesis: The killed bacteria affected a reduction in the bacterial viability of the same bacteria by a factor of 100, with no activity of the supernatant of the dead cells. Finally, it is in order to note that the effect described here is different in its mechanism from the post antibiotic effect (PAE) wherein bacteria do not resume growth for several hours following a transient exposure to antibiotics^29^,^30^: Whereas the PAE describes the delayed re-growth of *surviving* bacteria, we describe a post exposure phenomenon wherein *dead* bacteria affect the viability of living bacterial cells.
